# Effects of 18*β*-Glycyrrhetinic Acid on Fungal Protease-Induced Airway Inflammatory Responses

**DOI:** 10.1155/2018/6461032

**Published:** 2018-05-15

**Authors:** Yun Hee Kim, Dong Eon Kim, Seung-Hyo Lee

**Affiliations:** ^1^Graduate School of Medical Science and Engineering, Biomedical Research Center, KAIST Institute for the BioCentury, Korea Advanced Institute of Science and Technology (KAIST), Daejeon 34141, Republic of Korea; ^2^Korean Medicine Convergence Research Division, Korea Institute of Oriental Medicine (KIOM), Daejeon 34054, Republic of Korea

## Abstract

Airway epithelial cells secrete diverse inflammatory mediators in response to various stimuli. Thus, early regulation of immune responses in the airway epithelium is likely critical for the control of chronic inflammatory diseases. The purpose of the present study was to evaluate the effects of 18*β*-glycyrrhetinic acid (GA) on inflammatory responses generated in response to a fungal protease allergen that induces epithelial damage. To understand the underlying mechanisms, we also investigated the inhibitory effects of GA on the production of mitochondrial reactive oxygen species (ROS) in the human bronchial epithelial cell line BEAS2B. In this study, GA treatment reduced cytokine production and the human neutrophil cell line HL60 migration through decreased mitochondrial ROS production. In addition, GA significantly reduced inflammatory cell infiltration and cytokine levels in the bronchoalveolar lavage (BAL) fluid of fungal allergen-administered mice. Inhibitory effects of GA are dependent on the mitochondrial ROS/MAPK axis. Moreover, the effect of GA on the regulation of mitochondrial ROS depends on the expression of uncoupling protein-2 (UCP-2). Taken together, GA might represent a potential therapeutic agent for blocking inflammatory responses in airways.

## 1. Introduction

The airway epithelium is located at the interface between the host and environment and functions as the first line of defense against microorganisms, pollutants, and allergens [1, 2]. The physical barrier function of the airway epithelium depends on cellular integrity which is sustained by tight junction molecules. Various stimuli including potent allergens disrupt cellular integrity by cleaving the tight junction through their intrinsic enzymatic activity [3]. In addition, fungi, house dust mites, and/or pollen-originated allergens induce the secretion of other proteases and activate pattern recognition receptors (PRRs) in the airway epithelium. These activated PRRs, such as Toll-like receptor and protease-activated receptors, induce signaling cascades involved in immune responses [4]. Stimulated epithelia induce dendritic cells to mature and recruit various types of immune cells such as neutrophils, eosinophils, mast cells, and T cells to the airway. Thus, the airway epithelium not only acts as a physical barrier but also plays a key role in controlling airway inflammation [5–8].

Inhaled allergens induce the production of damage-associated molecular patterns (DAMPs) such as uric acid, adenosine triphosphate, and reactive oxygen species (ROS) to activate allergic immune responses [4]. Under physiological conditions, ROS are constantly generated while they also contribute to pathological conditions such as cardiovascular diseases, cancers, autoimmune diseases, and allergic diseases [9]. Although ROS are produced by several sources, including nicotinamide adenine dinucleotide phosphate (NADPH) oxidase and xanthine oxidase, mitochondrial ROS are thought to be critical for immune responses in several studies. Indeed, mitochondrial ROS induce innate immune responses through activation of inflammasomes, mitogen-activated protein kinases (MAPK), and retinoic acid-inducible gene-I- (RIC-I-) like receptors [10–12]. Thus, regulation of mitochondrial ROS is critical for controlling immune responses.

Licorice, the root of *Glycyrrhiza glabra*, has been used as a traditional medicinal plant for the treatment of various inflammatory diseases in many Asian and European countries [13]. Glycyrrhizin (GL) is the major bioactive compound of licorice and has been suggested to have various anti-inflammatory effects such as prevention of hepatic lipotoxicity, chronic hepatitis C, and lipopolysaccharide- (LPS-) induced inflammation [14–16]. GL exhibits bioactive properties through the functions of its biologically active metabolite, 18*β*-glycyrrhetinic acid (GA), which is produced via hydrolysis [14].

Although GA shows anti-inflammatory effects on various models, its effects on proteolytic allergen-mediated airway epithelial inflammation caused by direct tissue damage remain unknown. Accordingly, in this study, we aimed to evaluate the effects of GA on airway epithelial inflammation using a fungal protease allergen to induce epithelial damage, as previously described [17, 18]. Moreover, we also investigated the inhibitory effects of GA on the production of mitochondrial ROS in order to understand the underlying mechanisms. To this end, we found that GA suppresses mitochondrial ROS via an anion carrier protein, uncoupling protein-2 (UCP-2), and through the effects of GA on the activation of MAPK.

## 2. Materials and Methods

### 2.1. Cell Culture and Preparation of GA Solution

BEAS2B cells were purchased from the American Type Culture Collection (ATCC, Manassas, VA). The cells were grown in 100 mm tissue culture dishes (Corning, Corning, NY) in BEGM medium (Lonza, Basel, Switzerland) containing a bullet kit (Lonza) without antibiotics. The culture plates were maintained at 37°C in a humidified atmosphere of 95% air and 5% CO_2_.

GA was purchased from Sigma (St. Louis, MO; purity ≥ 97%) and dissolved in dimethylsulfoxide (DMSO; Sigma) to prepare 100 mM stock solutions. The 100% stock solution was diluted in cell culture medium at different concentrations and used immediately.

### 2.2. *Aspergillus* Protease, Inactive *Aspergillus* Protease, and MitoTEMPO Treatment

*Aspergillus* protease solution (>500 U/g, cat. number P6110, lot number SLBP2675V) was purchased from Sigma and was freshly diluted with serum-free media for immediate use. No live organisms were detected on our own microscopic analysis when the cells with *Aspergillus* protease solution were cultured. *Aspergillus* protease was tested at concentrations ranging from 10 to 1000 ng/ml. The enzyme activity of *Aspergillus* protease was 0.5 PU·mg^−1^ according to the manufacturer. Inactive protease was constructed by heat inactivation (100°C for 20 min), and proteolytic activity of protease was assessed using a protease activity assay kit (Abcam, Cambridge, MA). MitoTEMPO (Sigma) was dissolved in DMSO (Sigma) to generate 5 mM stock solutions and diluted in cell culture medium at different concentrations for immediate use.

### 2.3. MTS Assays for Analysis of Cell Viability

BEAS2B cells were plated at a density of 1 × 10^4^ cells/well in flat-bottomed 96-well plates containing BEGM medium. The cells were incubated for 24 h at 37°C in a humidified atmosphere of 95% air and 5% CO_2_. After incubation, cells were treated with different concentrations of *Aspergillus* protease diluted in cell culture medium. After another 1 h of incubation, cell viability was determined. To test the sensitivity to GA, BEAS2B cells were cultured in the medium containing various concentrations of GA and were incubated for 24 h. The viability of cells was assessed by the reduction of MTS (Promega, Madison, WI) to its formazan product, according to the manufacturer's instructions. Briefly, after removing the *Aspergillus* protease-stimulated medium, 200 *μ*l of medium with MTS solution was added to each well, and plates were incubated at 37°C for 4 h. The absorbance of the reaction at 490 nm was determined using a microplate fluorimeter (Molecular Devices, Sunnyvale, CA).

### 2.4. MCP-1 and IL-8 Cytokine Bead Assays (CBAs)

Cells were plated at a density of 0.5 × 10^6^ cells/well in flat-bottomed 6-well plates (Corning). Upon reaching confluence, the medium was changed to growth factor-free medium, and the plates were incubated for an additional 24 h after which the cells were stimulated with *Aspergillus* protease for 1 h. After washing the cells twice with medium, the cells were treated in the presence or absence of GA or MitoTEMPO for 23 h. The culture supernatants were collected and quantified using human CBA kits (BD Bioscience, Franklin Lakes, NJ) according to the manufacturer's instructions.

### 2.5. RNA Extraction and Real-Time Reverse Transcription Polymerase Chain Reaction (RT-PCR) Analysis

Total RNA was extracted from cultured cells using a Qiagen RNA extraction kit (Qiagen, Hilden, Germany). The concentration and quality of the RNA were determined using a NanoDrop 2000 spectrophotometer (Thermo Fisher Scientific, Waltham, MA). The cDNA was synthesized using a High-Capacity cDNA Reverse Transcription Kit (Applied Biosystems, Foster City, CA). Real-time quantitative PCR was performed using an ABI 7300 sequence detection system employing premade TaqMan probes; *MCP-1* probes (#Hs00234140), *ACTIN-β* probes (#Hs01060665), *eotaxin1* probes (#Hs00237013), *RANTES (CCL5)* probe (#Hs00982282), *IL-6* probe (#Hs00985639), *IL-1β* probe (#Hs01555410), *TNF-α* probe (#Hs01113624), *MIG-1* probe (#Hs00171065), and *IL-8* probe (#Hs00174103) (Thermo Fisher Scientific).

### 2.6. Immunofluorescence Confocal Microscopy

BEAS2B cells were cultured on glass-bottomed dishes (Thermo Fisher Scientific) and treated with different concentrations of *Aspergillus* protease for 1 h, followed by treatment with GA or MitoTEMPO for 23 h. After treatment, the cells were washed with PBS and incubated with CellROX orange at 37°C for 30 min or MitoSOX (Thermo Fisher Scientific) at 37°C for 20 min. The cells were then washed again with Hank's Balanced Salt Solution and mounted with PBS containing DRAQ5 (Cell Signaling Technology, Danvers, MA). After the final incubation for 5 min, the cells were analyzed using an FV10i confocal microscope (Olympus, Tokyo, Japan).

### 2.7. Western Blot Analysis

After treatment with *Aspergillus* protease and washing with PBS, BEAS2B cells were lysed in RIPA buffer (LPS Solution, Daejeon, Korea). The cell lysates (25 *μ*g/lane) were separated by electrophoresis and transferred onto polyvinylidene fluoride membranes. The membranes were blocked with blocking solution for 50 min at 23°C. After blocking, the membranes were incubated with rabbit anti-JNK, anti-phospho- (p-) JNK, anti-ERK1/2, anti-p-ERK1/2, anti-c-FOS, anti-c-JUN, anti-UCP-2, and anti-*β*-actin antibodies (Cell Signaling Technology; 1 : 1000 dilution) with gentle agitation overnight at 4°C. After incubation with the primary antibodies, the membranes were washed three times with TBST and incubated with biotinylated goat anti-rabbit IgG antibodies (Cell Signaling Technology) for 2 h at room temperature. The membranes were washed three times with the TBST, developed using Clarity Western ECL Blotting Substrate (Bio-Rad, Hercules, CA), and visualized using an ImageQuant LAS 4000 mini (GE Healthcare Life Science, Chicago, IL).

### 2.8. Small Interfering RNA (siRNA) Transfection

ON-TARGETplus siRNA against human UCP-2 was ordered from the predefined siRNA library of Qiagen. siRNA was used for transfection with Lipofectamine RNAi Max (Invitrogen, Carlsbad, CA) according to the manufacturer's protocol. Briefly, BEAS2B cells were transfected in Opti-MEM for 6 h, after which fresh BEGM was added to the cells and incubated for 15 h. The cells were then treated with *Aspergillus* protease for 1 h followed by incubation in serum-free medium. Protein and mRNA were isolated from these cells to analyze the levels of UCP-2 for evaluating UCP-2 expression.

### 2.9. Chemotaxis Assay

BEAS2B cells were stimulated with *Aspergillus* protease for 1 h and then treated with or without GA for 23 h. The supernatants were collected for chemotaxis assays using a QCM Chemotaxis 24-well (5 *μ*M) Cell Migration Kit (Millipore, Billerica, MA). HL60 cells at a density of 2 × 10^4^ cells/well were seeded in the upper wells of the kit, and culture supernatants were added to the lower wells. Following incubation for 2 h, media containing migrated cells from lower wells and detached cells from the bottom of the upper wells were dissolved in lysis buffer with dye. These samples were then transferred to 96-well plates, and the optimal density was measured using a microplate reader at 560 nm (Molecular Devices).

### 2.10. Mice

Wild-type C57BL/6 mice were bred in a specific pathogen-free animal facility. Female mice (6-7 weeks old) were used for this study. Animal care and experimental procedures were performed with the approval of the Animal Care Committee of Korea Advanced Institute of Science and Technology (KAIST, KA2015-25).

### 2.11. Induction of Experimental Allergic Inflammation

*Aspergillus* protease and OVA were purchased (Sigma-Aldrich) and reconstituted to 1 mg/ml using sterile PBS. *Aspergillus* protease and OVA were mixed at 1 : 10 (APO allergen) immediately before administration. Mice received four intranasal challenges every 4 days (days 0, 4, 8, and 12) with 50 *μ*l of APO allergen. GA was administered orally every day.

### 2.12. BAL Fluid and Quantification of Inflammatory Cells

Sixteen hours after the final intranasal challenge, mice were anesthetized with pentobarbital (Hanlim Pharma Co., Seoul, Korea) injection (0.15 ml/10 g body weight) intraperitoneally and intubated using a 20 G cannula. After intubation, the lung and trachea were washed via the cannula with PBS (1 ml, 4°C) for BAL fluid sampling. Total cells in BAL fluid were counted under a microscope, and 100 *μ*l BAL fluid was then stained with Hema 3 solution (Fisher HealthCare, Pittsburgh, PA). After estimating leukocyte proportions, the number of each cell type was calculated by multiplying the proportions by the total cell count. The remaining BAL fluid samples were used for CBA (BD Bioscience).

### 2.13. Statistical Analysis

Statistical analysis was conducted using Prism 6.0 software with one-way analysis of variance for multiple comparisons (GraphPad Software Inc., San Diego, CA). The results are shown as the means ± standard errors of the means (SEMs) of three independent experiments. In all cases, differences with *P* values of less than 0.05 were considered significant.

## 3. Results

### 3.1. Effects of *Aspergillus* Protease on the Production of Inflammatory Mediators

**Figure 1:**
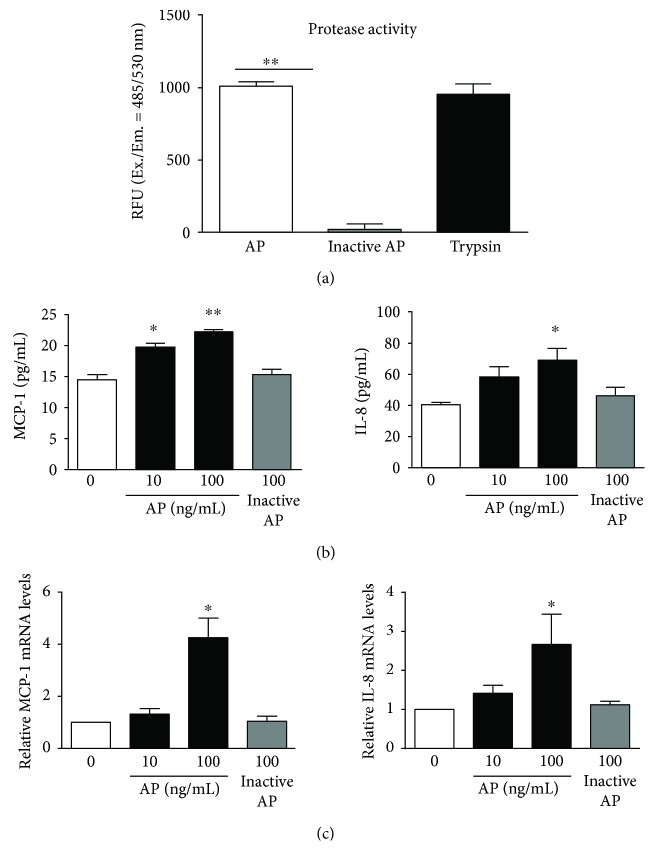
Effects of *Aspergillus* protease on the production of inflammatory mediators. (a) The activity of protease was assessed using a protease activity kit. Trypsin was used as a positive control group. (b) MCP-1, IL-8, and MIG cytokine levels were quantified in BEAS2B cells treated with *Aspergillus* protease or inactive protease for 1 h using CBA. (c) *MCP-1* and *IL-8* mRNA expression was quantitated in BEAS2B cells treated with *Aspergillus* protease. Gene expression levels were normalized to the expression of *β*-actin mRNA (0: control group; without *Aspergillus* protease treatment). ^∗^*P* < 0.05 and ^∗∗^*P* < 0.01 compared with the control (0) group.

Proteolytic activity of inactive protease was significantly decreased in comparison with that of *Aspergillus* protease (Figure 1(a)). To investigate the effects of *Aspergillus* protease on inflammatory responses, the production of interleukin- (IL-) 1*β*; tumor necrosis factor- (TNF-) *α*; IL-8; regulated on activation, normal T cell expressed and secreted (RANTES); monocyte chemotactic protein- (MCP-) 1, eotaxin-1, and IL-6 was assessed in inactive protease- or *Aspergillus* protease-stimulated BEAS2B cells. After stimulation with *Aspergillus* protease (10 or 100 ng/ml), the cells exhibited increased expression of MCP-1 and IL-8 proteins (Figure 1(b)). We next quantified the mRNA expression of *IL-1β*, *TNF-α*, *IL-8*, *RANTES*, *MIG*, *MCP-1*, eotaxin-1, and *IL-6*. Although the levels of other inflammatory mediators were not significantly elevated (data not shown), *MCP-1 mRNA* was increased following *Aspergillus* protease treatment for 1 h. In the case of *IL-8*, mRNA expression was increased after 30-minute incubation with *Aspergillus* protease and elevated *IL-8* mRNA level was decreased after another 30 minutes. According to previous studies, cytokine mRNA levels, such as for IL-8, could respond rapidly to environmental changes and stimulation and rapidly turn over [19, 20] (Figure 1(c)). Therefore, these results indicate that *Aspergillus* protease induces expression of inflammatory mediators in the airway epithelium.

### 3.2. Effects of *Aspergillus* Protease on Intracellular and Mitochondrial ROS Production

**Figure 2:**
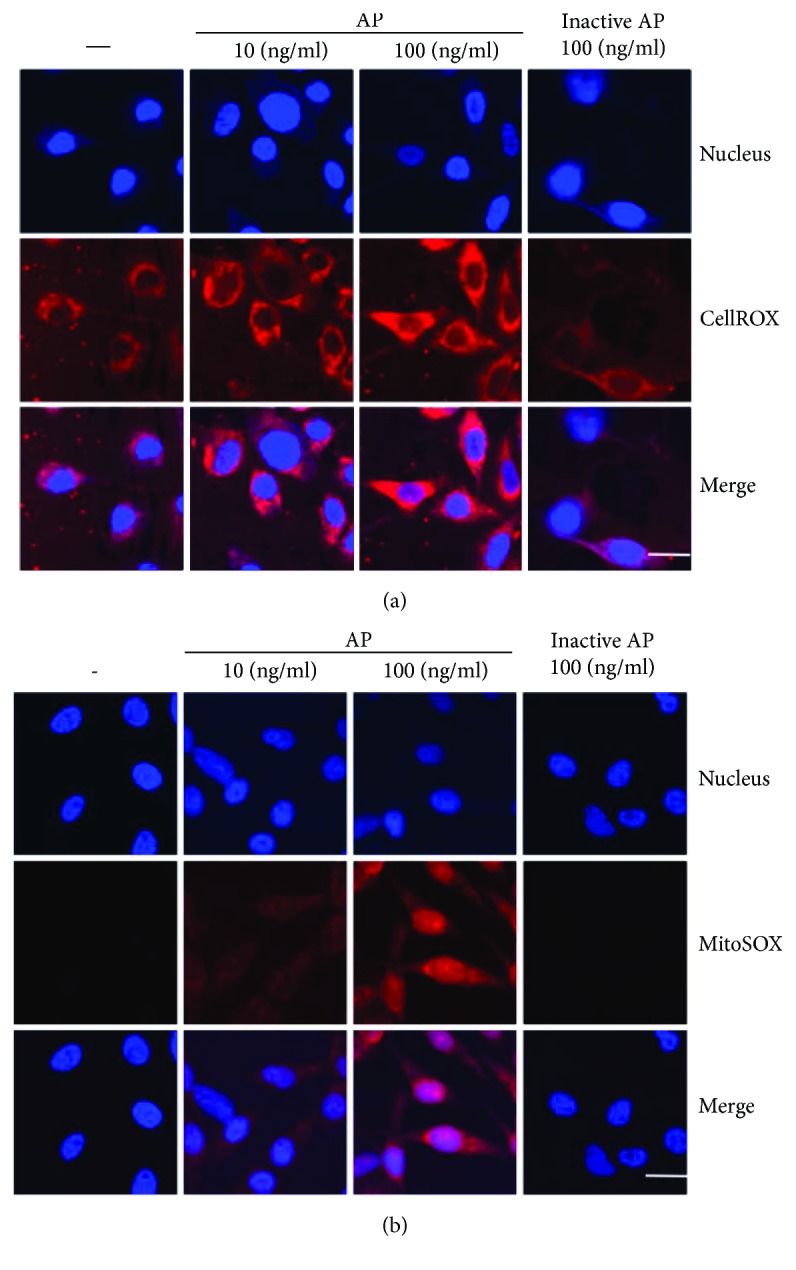
Effects of *Aspergillus* protease on intracellular and mitochondrial ROS production. (a) The levels of intracellular ROS in *Aspergillus* protease-treated cells were evaluated using CellROX. (b) BEAS2B cells were incubated with the mitochondrial ROS indicator MitoSOX and analyzed using confocal microscopy (magnification 130x, scale bar = 20 *μ*m) after stimulation with *Aspergillus* protease.

ROS are critical signaling molecules for inflammatory responses [21]; therefore, we measured the levels of *Aspergillus* protease-induced intracellular ROS production by staining cells with CellROX. The levels of intracellular ROS were increased in response to *Aspergillus* protease in a concentration-dependent manner (Figure 2(a)). It is known that mitochondrial ROS is the major source of intracellular ROS [10]. Thus, we wanted to quantify the levels of mitochondrial ROS after *Aspergillus* protease stimulation. As shown in Figure 2(b), *Aspergillus* protease-treated BEAS2B cells produced increased levels of mitochondrial ROS in a dose-dependent manner. Thus, these results indicate that both intracellular and mitochondrial ROS levels are markedly enhanced following *Aspergillus* protease stimulation of BEAS2B cells.

### 3.3. The Effects of GA on the Production of Inflammatory Mediators, ROS, Antioxidant Enzymes, and UCP-2

**Figure 3:**
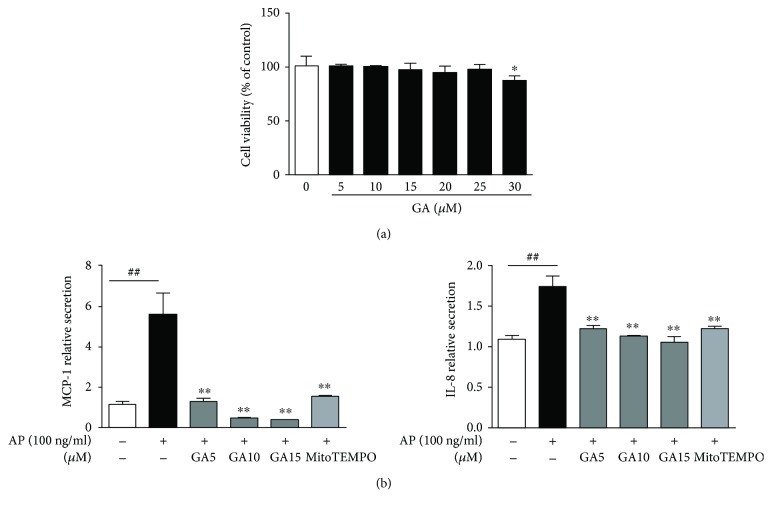
Inhibitory effects of GA on cell viability and the production of inflammatory mediators. (a) BEAS2B cells were incubated for 24 h in the presence of varying concentrations of GA. Cell viability was determined using the MTS assay as described in Materials and Methods. (b) MCP-1 and IL-8 cytokine levels were quantified with CBAs in GA-treated BEAS2B cells after *Aspergillus* protease stimulation. ^∗^*P* < 0.05 and ^∗∗^*P* < 0.01 compared with the *Aspergillus* protease stimulation-only group, ^##^*P* < 0.01 compared with the control group.

**Figure 4:**
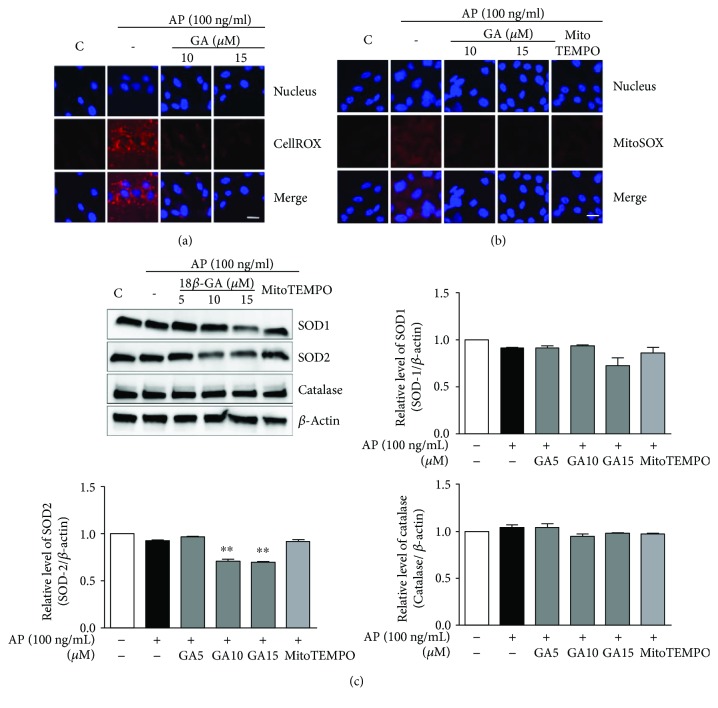
Inhibitory effects of GA on the production of intracellular and mitochondrial ROS. (a) The level of intracellular ROS following GA treatment and *Aspergillus* protease stimulation was evaluated using CellROX. (b) Mitochondrial ROS were evaluated using MitoSOX and analyzed with confocal microscopy after stimulation with *Aspergillus* protease (magnification 130x, scale bar = 20 *μ*m). (c) SOD-1, SOD-2, and catalase levels were determined in BEAS2B cells after GA treatment following *Aspergillus* protease stimulation by Western blot analysis. *β*-Actin was used as an internal control. Images were quantified using ImageJ. ^∗∗^*P* < 0.01 compared with the *Aspergillus* protease stimulation-only group.

**Figure 5:**
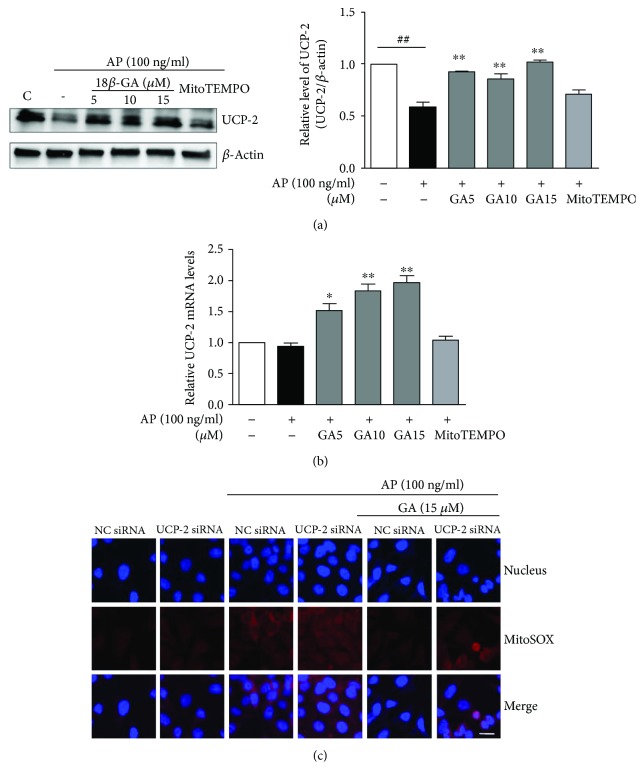
Effects of GA on the expression of UCP-2 and on mitochondrial ROS in the context of UCP-2 knockdown. (a) The UCP-2 protein level was determined by Western blot analysis in BEAS2B cells after GA treatment of *Aspergillus* protease-stimulated cells. Images were acquired using LAS and quantified with ImageJ. *β*-Actin was used as an internal control. (b) *UCP-2* mRNA expression levels were normalized to the expression of *β*-actin mRNA as a control. (c) The level of mitochondrial ROS in GA-treated BEAS2B cells with UCP-2 knockdown under *Aspergillus* protease stimulation. NC siRNA: negative-control siRNA-transfected cells; UCP-2 siRNA: UCP-2 siRNA-transfected cells (magnification 130x, scale bar = 20 *μ*m). ^∗^*P* < 0.05 and ^∗∗^*P* < 0.01 compared with the *Aspergillus* protease stimulation-only group, ^##^*P* < 0.01 compared with the control group.

The anti-inflammatory effects of GA have previously been shown in various models [14–16]. Therefore, we evaluated the effects of GA on inflammatory mediators in BEAS2B cells to determine the potential of this compound for therapeutic studies. The cytotoxic effects of GA have been studied as a primary screening for anti-inflammatory effects. Treatment of GA at different concentrations such as 5, 10, 15, 20, and 25 *μ*M for 24 h resulted in no cytotoxic effects. It exhibited 13.4% reduced cell viability at 30 *μ*M (Figure 3(a)). In the presence of GA, the production of MCP-1 and IL-8 proteins was reduced, although the mRNA levels were not changed (Figure 3(b), data not shown). Thus, to understand the molecular mechanisms underlying the effect of GA on inflammatory mediators, we quantified *Aspergillus*protease-induced intracellular and mitochondrial ROS after addition of GA (10 or 15 *μ*M) and found that intracellular ROS production was significantly reduced in BEAS2B cells (Figure 4(a)). More importantly, the levels of mitochondrial ROS were also diminished by GA in a manner similar to the scavenger of mitochondrial superoxide, MitoTEMPO (Figure 4(b)). Because of the effects of GA on intracellular and mitochondrial ROS production, we next wanted to examine whether GA-mediated suppression of ROS production is regulated by antioxidant enzymes. To this end, we first treated BEAS2B cells with GA (5, 10, or 15 *μ*M) for 24 hours after a 1-hour stimulation with *Aspergillus* protease. However, we found that the protein levels of superoxide dismutase-1 (SOD-1), SOD-2, and catalase were not increased in the GA- and *Aspergillus* protease-treated group (Figure 4(c)). It indicated that the effect of GA on mitochondrial ROS did not depend on SOD-2. Moreover, decreased expression of SOD-2 is supposed to be the consequence of the decreased mitochondrial ROS in GA-treated group. Therefore, we next assessed the expression of UCP-2, which is known to be a negative regulator of mitochondrial ROS production. As seen in Figure 5, *Aspergillus* protease decreases the expression of UCP-2 at both the mRNA and protein levels, and the decreased UCP-2 expression level was reversed by GA treatment (Figures 5(a) and 5(b)). Therefore, these results indicate that *Aspergillus* protease induces mitochondrial ROS through suppression of UCP-2, effects which are reversed by treatment with GA.

To determine whether the GA-mediated increase in expression of UCP-2 is directly associated with mitochondrial ROS production, we silenced the *UCP-2* gene and assessed the effects of GA on mitochondrial ROS in the absence of UCP-2. The inhibitory effects of GA on *Aspergillus* protease-mediated mitochondrial ROS production were diminished in UCP-2-knockdown cells (Figure 5(c)). These results suggest that the inhibitory function of GA against *Aspergillus* protease-induced mitochondrial ROS production is mediated by UCP-2.

### 3.4. Inhibitory Effects of GA on HL60 Cell Migration Are Mediated through Activation of MAPK and Activator Protein- (AP-) 1

**Figure 6:**
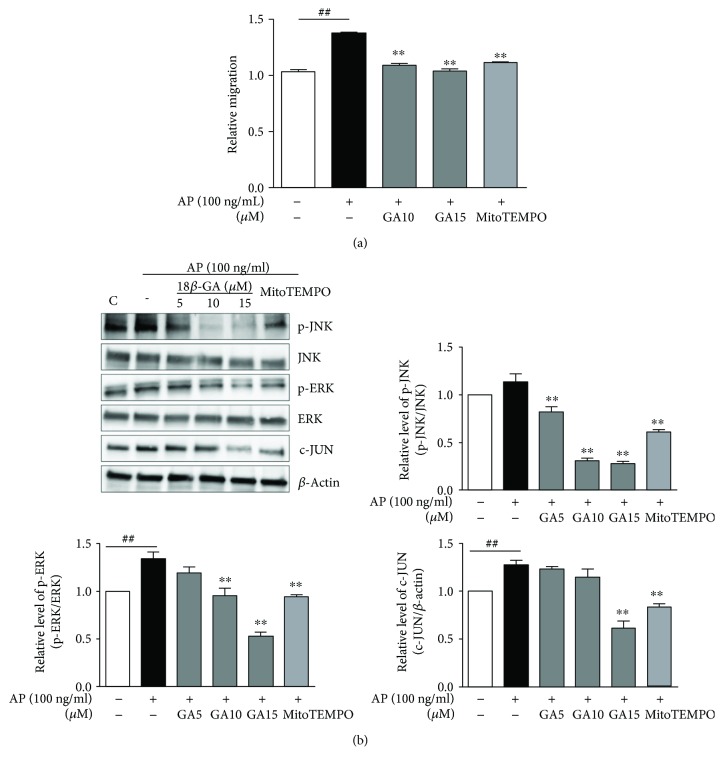
Inhibitory effects of GA on HL60 cell migration through suppression of MAPK and AP-1. (a) Migration of HL60 cells was inhibited by GA in BEAS2B cells stimulated with *Aspergillus* protease. Representative data of three independent experiments is presented. ^##^*P* < 0.01 compared with the control group (control group; neither *Aspergillus* protease stimulated nor GA treated), ^∗∗^*P* < 0.01 compared with *Aspergillus* protease stimulation without GA. (b) Phosphorylation of JNK and ERK1/2 and expression of c-JUN were determined by Western blot analyses. Images were acquired using LAS and quantified with ImageJ. *β*-Actin was used as an internal control. ^##^*P* < 0.01 compared with the control group (control group; neither *Aspergillus* protease stimulation nor GA treatment).

Because IL-8 and MCP-1 are well-known neutrophil chemoattractants [22], we next investigated the migration of HL60 human neutrophils using supernatants from BEAS2B cells after *Aspergillus* protease stimulation and GA treatment. Compared with the *Aspergillus* protease-stimulated group, GA inhibited the HL60 cell migration in the GA group (Figure 6(a)). This effect was similar to that induced by the control, MitoTEMPO. To elucidate the molecular mechanisms underlying these effects, we evaluated the activation of c-Jun N-terminal kinase (JNK), extracellular signal-regulated kinase (ERK), and AP-1 following GA treatment. Western blot analyses showed significantly reduced phosphorylation of JNK and ERK resulting in decreased activation of AP-1 following GA and MitoTEMPO treatment (Figure 6(b)). Taken together, these data indicate that GA may inhibit the production of inflammatory mediators via mitochondrial ROS- and MAPK-dependent mechanisms in the airway epithelial cells.

### 3.5. Inhibitory Effects of GA on Inflammatory Responses in an *Aspergillus* Protease-Mediated Lung Inflammation Model

**Figure 7:**
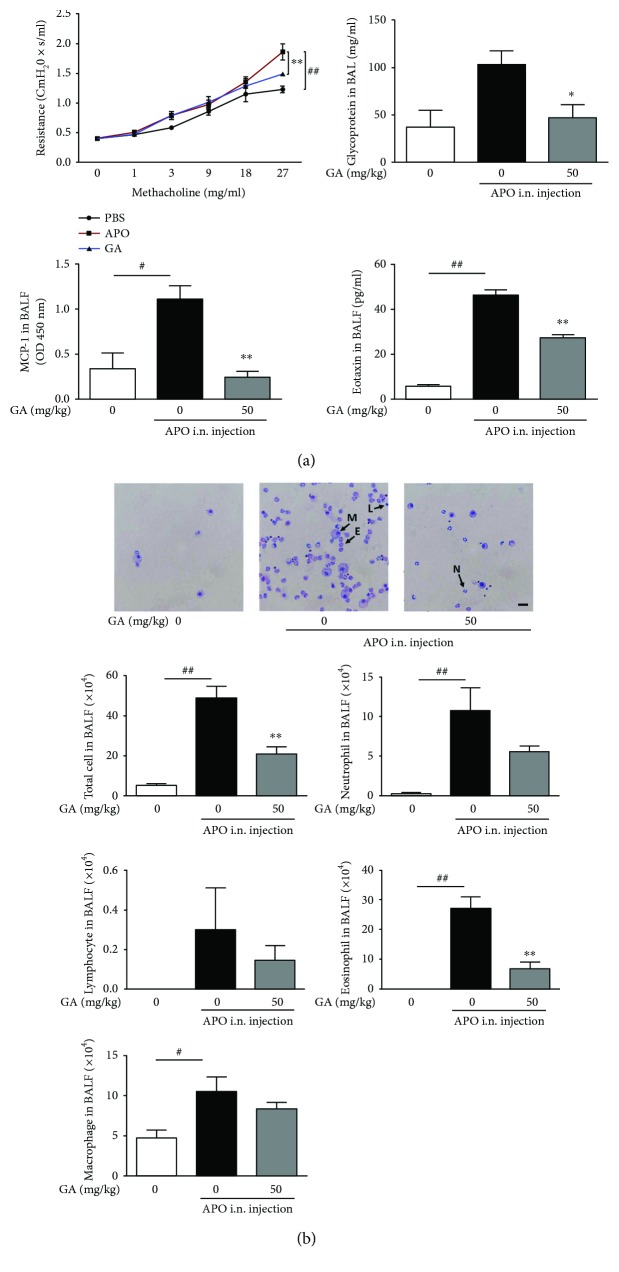
Inhibitory effects of GA on *in vivo* inflammatory responses. AHR was measured with the flexiVent system using the methacholine test. Cells in BALF were precipitated and stained with Giemsa, and cytokine levels were assessed by ELISA and CBA. (a) Inhibitory effects of GA on AHR, glycoprotein secretion, and chemokines in BAL fluid. (b) Inhibitory effects of GA on infiltration of inflammatory cells in BAL fluid (magnification 40x, scale bar = 100 *μ*m). M: macrophage; E: eosinophil; N: neutrophil; L: lymphocyte. Data are presented as the mean ± SEM (*n* = 3). ^∗^*P* < 0.05 and ^∗∗^*P* < 0.01 compared with APO groups without GA treatment, ^#^*P* < 0.05 and ^##^*P* < 0.01 compared with the no-APO treatment group.

To further assess the therapeutic potential of GA, the *in vivo* inhibitory effects of GA on inflammatory cell infiltration were investigated utilizing the *Aspergillus* protease-induced lung inflammation model. In this model, wild-type C57BL/6 mice are challenged intranasally with *Aspergillus* protease and chicken egg ovalbumin (OVA; APO) resulting in airway hyperresponsiveness (AHR) and inflammatory cell infiltration into bronchoalveolar lavage (BAL) fluid [23]. For these studies, APO allergen is intranasally administered every four days for a total of four times with or without daily oral administration of GA (50 mg/kg). Compared with the PBS-challenged negative control, the APO-challenged group showed increased AHR and glycoprotein secretion into BAL fluid, effects that were partially reversed by GA treatment (Figure 7(a)). Because GA treatment inhibits the production of the neutrophil chemoattractant MCP-1 by BEAS2B cells, we next quantified MCP-1 and the eosinophil chemoattractant eotaxin-1 in BAL fluid of treated mice. As shown in Figure 7(a), both MCP-1 and eotaxin-1 were downregulated in the BAL fluid following GA treatment. Moreover, inflammatory cell recruitment was particularly diminished in the GA-treated group (Figure 7(b)). Therefore, these data demonstrate that GA exhibits inhibitory effects on airway inflammation, which are likely mediated through decreased recruitment of inflammatory cells.

## 4. Discussion

In the epithelium, persistent, chronic inflammation occurs through the recruitment and activation of inflammatory cells [5, 24]. Therefore, targeting airway epithelium-mediated immune responses is a potential therapeutic strategy to regulate acute and ongoing inflammation. In this study, we investigated the production of inflammatory mediators and neutrophil chemotaxis to assess the inhibitory effects of GA on epithelial cells following *Aspergillus* protease stimulation. Our results show that GA reduces cytokine production, HL60 cell migration, cellular and mitochondrial ROS production, and MAPK activation in BEAS2B bronchial epithelial cells. In addition, GA reduces inflammatory cell infiltration and cytokine production in BAL fluid of *Aspergillus* protease-administered mice. Thus, our findings indicate that in the range of concentrations tested, GA regulates airway inflammation through inhibition of proinflammatory cytokine-induced chemotaxis via suppression of the mitochondrial ROS/MAPK axis and related signaling pathways.

It is well known that *Aspergillus* protease induces multifunctional inflammatory cytokines, such as MCP-1 and IL-8. MCP-1 stimulates migrating macrophages and monocytes [25], and MCP-1 and IL-8 also activate and recruit neutrophils [22]. Thus, regulation of MCP-1 and IL-8 levels contributes to the control of the persistent immune response, and the *Aspergillus* protease-induced secretion of these molecules was significantly decreased following GA treatment. These results show that GA functions to control airway inflammation via inhibiting recruitment of neutrophils and T lymphocytes.

Under pathological conditions, disproportionate generation of ROS causes inflammation and tissue damage [26]. Mitochondria are known to be one of the major sources of intracellular ROS [27] and have functional significance in inflammation. In addition, recent studies have shown that mitochondrial ROS play important roles in the production of inflammatory mediators and immune modulation in the innate immune system [10, 26, 28–30]. Moreover, ROS-induced MAPK activation plays a key role in signal transduction in immune responses to various antigens including LPS [30–33]. In our study, GA was found to inhibit intracellular and mitochondrial ROS production, as well as MAPK activation in a manner similar to the mitochondrial ROS scavenger, MitoTEMPO. MitoTEMPO is a recently suggested perfect candidate for scavenging mitochondrial ROS which has functioned as mitochondria-targeted SOD [30]. In our experiment, GA shows to be more effective in decreasing mitochondrial ROS. Our results also show that decreased mitochondrial ROS following GA treatment of *Aspergillus* protease-stimulated cells reduces JNK and ERK activation as well as AP-1 expression. Therefore, GA may regulate MAPK activation via suppression of mitochondrial ROS in *Aspergillus* protease-stimulated BEAS2B cells. Based on these results, we propose that GA regulates expression of MCP-1 and IL-8 proteins via suppression of the mitochondrial ROS/MAPK axis.

To elucidate the mechanisms through which GA suppresses intracellular and mitochondrial ROS, we evaluated the levels of antioxidant enzymes previously reported to be elevated in response to GA treatment [34]. However, in our study, the expression levels of antioxidant enzymes such as SOD-1, SOD-2, and catalase were not increased. Moreover, the expression level of SOD-2 was decreased depending on the concentration of GA. It is possible that the necessity of SOD is reduced because GA effectively decreases superoxide anion. More importantly, when the expression of UCP-2 was assessed, an anion carrier protein located in the mitochondrial inner membrane functions to reduce membrane potential and modulate superoxide anion production via proton leakage, thereby protecting against oxidative stress [35, 36]. GA treatment was found to increase UCP-2 expression following *Aspergillus* protease stimulation. In addition, the inhibitory effect of GA on mitochondrial ROS was abrogated in the absence of UCP-2 expression. These results indicate that GA reduces mitochondrial ROS via upregulation of UCP-2 rather than through modulation of antioxidant enzymes. Furthermore, GA also inhibits *in vivo* epithelial inflammation including infiltration of neutrophils, eosinophils, and lymphocytes into BAL fluid in the fungal protease-mediated airway inflammation model.

In summary, our study demonstrates that GA is a potent inhibitor of *Aspergillus* protease-induced expression of inflammatory mediators including MCP-1 and IL-8. In addition, *in vivo* epithelial inflammation induced by *Aspergillus* protease stimulation is inhibited by GA. These inhibitory effects are dependent on the mitochondrial ROS/MAPK axis and regulation of UCP-2 expression. Therefore, GA may represent a potential effective therapeutic agent for blocking inflammatory responses in airways. However, further studies using UCP-2-knockout models will be required to fully elucidate the mechanism underlying GA's effects on mitochondrial ROS regulation in airway epithelial inflammation and how GA might cause this increase in UCP-2.
